# Comparative Metabolomics and Transcriptome Analysis Reveal the Fragrance-Related Metabolite Formation in *Phoebe zhennan* Wood

**DOI:** 10.3390/molecules28207047

**Published:** 2023-10-12

**Authors:** Hanbo Yang, Fang Wang, Wenna An, Yunjie Gu, Yongze Jiang, Hongying Guo, Minhao Liu, Jian Peng, Bo Jiang, Xueqin Wan, Lianghua Chen, Xiong Huang, Fang He, Peng Zhu

**Affiliations:** 1 Forestry Ecological Engineering in the Upper Reaches of the Yangtze River Key Laboratory of Sichuan Province, National Forestry and Grassland Administration Key Laboratory of Forest Resource Conservation and Ecological Safety on the Upper Reaches of the Yangtze River, Rainy Area of West China Plantation Ecosystem Permanent Scientific Research Base, College of Forestry, Sichuan Agricultural University, Chengdu 611130, China; yanghanbo6@sicau.edu.cn (H.Y.); 2020204014@stu.sicau.edu.cn (F.W.); 202001218@stu.sicau.edu.cn (W.A.); 72076@sicau.edu.cn (Y.J.); wanxueqin@sicau.edu.cn (X.W.); chenlh@sicau.edu.cn (L.C.); 14880@sicau.edu.cn (X.H.); 14686@sicau.edu.cn (F.H.); 2 Sichuan Academy of Forestry, Sichuan Key Laboratory of Ecological Restoration and Conservation for Forest and Wetland, Chengdu 610081, China; zhongli6@126.com (M.L.); 2020304092@stu.sicau.edu.cn (J.P.); 3 Sichuan Academy of Grassland Sciences, Chengdu 610041, China; yanghanbo6@163.com (H.G.); 2020304074@stu.sicau.edu.cn (B.J.); 4 Du Fu Thatched Cottage Museum, Chengdu 610001, China

**Keywords:** *Phoebe zhennan*, metabolomics, fragrance, heartwood, GC-MS, transcriptome

## Abstract

Nanmu (*Phoebe zhennan*) has a unique fragrance and is a high-quality tree species for forest conservation. The types and contents of volatile compounds in different tissues of nanmu wood are different, and the study of its volatile metabolites can help us to understand the source of its fragrance and functions. In order to explore the metabolites related to the wood fragrance of nanmu and to find out the unique volatile substances in the heartwood, gas chromatography–mass spectrometry (GC-MS) was performed to analyze the non-targeted metabolomics in five radial tissues from the sapwood to the heartwood of nanmu. A total of 53 volatile metabolites belonging to 11 classes were detected in all tissues, including terpenes, aromatic hydrocarbons, organoheterocyclics, phenols, esters, organic acids, alcohols, alkaloids, alkane, indoles derivatives, and others. And most of the volatile metabolites were identified for the first time in nanmu wood. Among them, terpenes and aromatic hydrocarbons were the main volatile components. In addition, 22 differential metabolites were screened from HW and SW, HW, and TZ via metabolomic analysis. Among these DAMs, three volatile metabolites (cadinene, a sesquiterpenoid; *p*-cymene, a monoterpenoid; 1,3,5-triisopropylbenzene, an aromatic hydrocarbon) contributed heavily to the characteristic fragrance of the heartwood. Additionally, the expression of transcripts showed that the unigenes in the terpenoid biosynthesis pathway were especially up-regulated in the SW. Therefore, we speculated that fragrance-related metabolites were synthesized in SW and then deposited in heartwood during sapwood transformed to heartwood. The expression levels of transcription factors (e.g., WRKY, C2H2, NAC) acted as the major regulatory factors in the synthesis of terpenoid. The results lay the foundations for further studies on the formation mechanism of fragrance components in nanmu wood and also provide a reference for the further development and utilization of nanmu wood.

## 1. Introduction

Nanmu (*Phoebe zhennan* S. Lee et F.N. Wei) belongs to the Lauraceae family and Machilus Nees genus and is an evergreen broad-leaved tree. It is a rare species that is endemic to China and mainly distributed in Sichuan, Guizhou, and Hubei [1,2]. Its trunk is straight and graceful, and its crown is tight, making it a famous garden ornamental tree species [3]. The wood is known as “golden-thread nanmu” due to its dense texture, strong corrosion resistance, and metallic luster, which was the main species of nanmu in the Forbidden City in China [3,4,5,6]. One of the most important characteristics of nanmu is that its wood has a fragrance, which is an important breeding characteristic that reflects the economic and cultural value of nanmu [2]. Furthermore, *P. zhennan* is a source of wood essential oils, containing volatile organic compounds (VOCs) with high economic value in the spice and perfume industries [7]. The essential oil from the *P. zhennan* woods had good biological activity for potential product development [8]. Hence, exploration of the fragrance-related metabolites and the mechanism of the biosynthesis pathway in *P. zhennan* could enhance its production and overall quality in future breeding programs for wood improvement.

The xylem of most wood plants consists of three tissues: the sapwood, the transition zone, and the heartwood [9]. The sapwood is composed of living cells with physiological activity and mainly affects transport and storage for plant growth [10]. The darkest part is the heartwood, which is usually considered the most important part of the trunk. In the process of heartwood formation, the accumulation of secondary metabolites (volatile metabolites), such as terpenoids (sesquiterpenoids, monoterpenes, and diterpenoids, etc.), is endowed with special fragrance for heartwood [11,12,13]. Wood volatile metabolites contain a variety of chemical components, and these chemical components not only emit a variety of odors but also have certain healthcare functions and medicinal value. The new section of wood in *Phoebe* spp. has a fragrance and is widely used in spice production, perfume production, health care, and other industries. At present, scholars have studied the components of nanmu (*Phoebe zhennan*) essential oil. For example, Xie et al. [7] compared the essential oil content and chemical composition of modern and ancient wood of nanmu. Ding et al. [14] studied the essential oil in the leaves of *Phoebe bournei* and found that sesquiterpenes were the main components. Han et al. [15] studied the volatile compounds in the stem and xylem of *P. bournei*, which showed that sesquiterpenes were the main volatile organic compounds. Yang et al. [11] reported that monoterpenoid and sesquiterpenes were the main fragrance-related metabolites in *Phoebe hui* wood. Metabolomics involves the qualitative or quantitative analysis of substances in organisms at specific times and under specific conditions to explain the response of plants to the external environment [16]. GC-MS is a useful tool in metabolomic studies to identify small molecular metabolites and fragrance-related volatile compounds in plants, including terpenoids, phenols, and fatty acids [17,18]. In recent years, this technique has been widely used in the identification of wood volatile components. For example, Peng et al. [19] analyzed the volatile fragrant components of agarwood using GC-MS and identified 484 volatile compounds, among which aromatic compounds were the main chemical components of fragrance. Liu et al. [20] carried out a GC-MS analysis on the volatile substances of wood products and found that esters and aromatic hydrocarbons were the main causes of the odor. Many studies identified that wood extract is the main source of wood fragrance, so the study of nanmu extract is the basis of studying the volatile metabolites of nanmu. Therefore, it is feasible to analyze the volatile components of nanmu wood using GC-MS.

Secondary metabolites are the final products that directly reflect phenotypic and functional changes produced by the plant metabolism driven by gene regulation [2]. Previous studies have demonstrated that heartwood formation and the biosynthesis of secondary metabolites are mainly genetically determined [11]. As the main components of fragrance-related metabolites, there have been many studies on the biosynthesis pathway of terpenoids in plants. In vivo, terpenoids can be synthesized via two different pathways, the mevalonate (MVA) pathway and the 2-C-Methyl-D-erythritol-4-phosphate (MEP) pathway [21]. Terpenoids are synthesized by terpene synthases (TPSa) via prenyl diphosphates: geranly-PP (GPP) for monoterpenes, farnesyl-PP (FPP) for sesquiterpenes, and geranylgeranyl-PP (GGPP) for diterpenes in plants [22]. Based on joint analysis of the transcriptome and metabolism, Yang et al. [11] screened the pathway genes (UEX, GN1_2_3, and AMY, etc.) and transcription factors (MYB, WRKY, and C2H2, etc.) in terpenoid biosynthesis. HMGR, 1-deoxy-D-xylulose-5-phosphate synthase (DXS), 1-deoxy-D-xylulose-5-phosphate reductoisomerase (DXR), isoprene pyrophosphate synthase, and TPS have been confirmed as the key genes encoding the key enzymes in the biosynthetic pathways of terpenoids [21]. Celedon et al. [23] found that the genes encoding (Z)-specific P450s contribute to sandalwood oil formation in heartwood with upstream genes of sesquiterpene biosynthesis. Yeh et al. [24] found that the highly expressed TPS-encoding, GGPS-encoding, and FPS-encoding genes in the MVA pathway and MEP pathway in the transition zone contributed to a gradual increase in the accumulation of terpenoids from the transition zone towards heartwood in *Taiwania cryptomerioides*. Transcription factors are involved in the regulation of multiple key genes in terpenoid biosynthesis; for instance, AP2/ERF, bHLH, MYB, NAC, WRKY, and bZIP have been determined to be involved in the biosynthesis of terpenoids [25]. Although the significance of the volatile metabolites and related genes has been studied in some wood plants, several questions remain regarding the formation and regulatory mechanisms of the fragrance-related metabolites in *P. zhennan* wood.

The aim of this study is to utilize GC-MS technology and RNA-seq from different xylem tissues, representing the heartwood formation process of *P. zhennan*. Then, combined with metabolomics and transcriptome analysis, we explore the volatile aroma compounds of *P. zhennan* wood to find out the volatile unique substances and the key genes in heartwood fragrance formation. The findings of this study will provide valuable information on the *P. zhennan* breeding process and provide a possibility for the comprehensive utilization of *P. zhennan* wood.

## 2. Results

### 2.1. Metabolic Profiles

**Figure 1:**
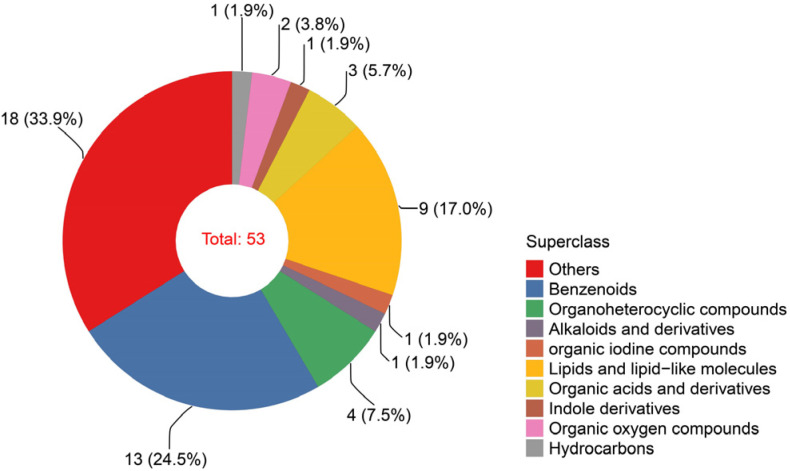
Classification of the identified metabolites detected in all samples.

A total of 53 volatile metabolites were detected in all samples, of which 35 were annotated in the public database. These metabolites were annotated to the HMDB, which were matched and classified into nine superclasses, including 13 benzenoids, 9 lipids and lipid-like molecules, 4 organoheterocyclic compounds, 3 organic acids and derivatives, 2 organic oxygen compounds, 1 alkaloid and derivative, 1 organic iodine compound, 1 indole derivative, and 1 hydrocarbon (Figure 1). Additionally, these metabolites were divided into 10 classes, including 8 terpenes, 8 aromatic hydrocarbons, 5 organoheterocyclics, 5 phenols, 3 esters, 3 organic acids, 2 alcohols, 1 alkaloid, 1 alkane, and 1 indole derivative (Figure S1, Table S1). Lipids and lipid-like molecules (e.g., terpenes) and benzenoids (e.g., aromatic hydrocarbons) may be the main sources of the fragrance of *Phoebe zhennan* wood.

### 2.2. Principal Component Analysis (PCA) and Hierarchical Cluster Analysis (HCA) of All Tissues

**Figure 2:**
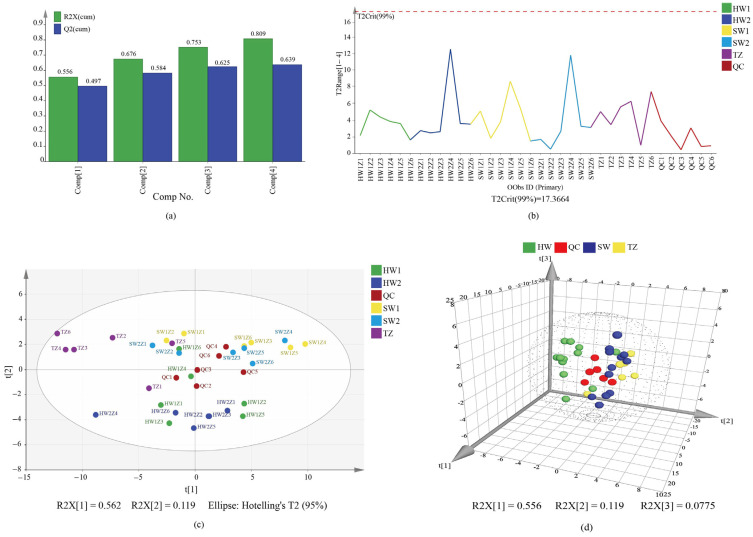
Principal component analysis (PCA) of the metabolite distribution among sapwood, transition zone, and heartwood from *Phoebe zhennan* wood. (**a**) Summary of fit of all samples. The column represents the explained ratio of principal components. (**b**) Hotelling’s T^2^ range line plot of all samples with 99% confidence interval. TZ: the transition zone, HW1: the heartwood part far away from the TZ, HW2: the heartwood part located near the TZ, SW1: the inner sapwood of the sapwood section far away from the TZ, SW2: the outer edge of the sapwood part near the TZ, QC: the quality control. HW1Z1-HW1Z6, HW2Z1-HW2Z6, SW1Z1-SW1Z6, SW2Z1-SW2Z6, TZ1-TZ6, and QC1-QC6 represented the six biological replicates of HW1, HW2, SW1, SW2, TZ, and QC, respectively. (**c**) PCA score scatter plot of all samples with QC under 95% confidence interval. (**d**) Three-dimensional PCA plot of all samples with QC. HW1 and HW2 combined into HW (heartwood); SW1 and SW2 combined into SW (sapwood).

Principal component analysis (PCA) was carried out to determine the different accumulation of secondary metabolites among the five tissues (Figure 2). The four components in the PCA explained 80.9% of the total variance (Figure 2a). Additionally, all the samples remained within the 99% confidence interval, according to Hoteling’s T^2^ analysis (Figure 2b). The results of the PCA showed that quality control samples (QC) were distributed in the middle of samples of five tissues, indicating that the stability of the instrument was high (Figure 2c). Meanwhile, the PCA diagram also showed that the separation trend of heartwood (HW), sapwood (SW), and transition zone (TZ) tissue groups was larger, and the difference within groups was small (Figure 2c,d). This suggested that the data had good reliability.

**Figure 3:**
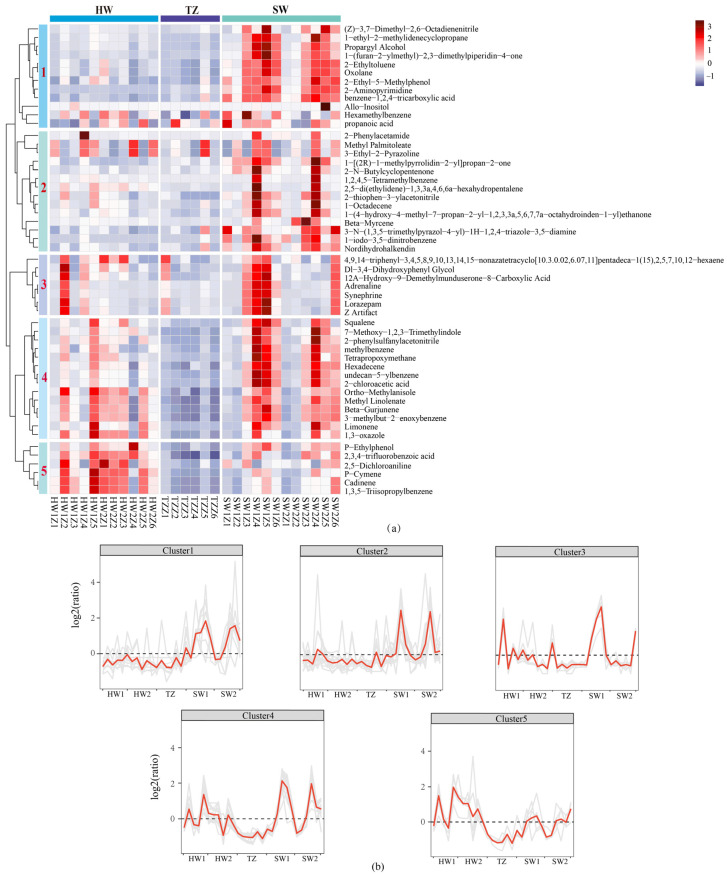
Hierarchical cluster analysis of metabolites in all tissues. (**a**) Metabolite heat map with hierarchical clustering. The sample grouping is represented by the horizontal axis of the heat map, and the metabolites are represented by the vertical axis. Different colors represent the standardized values of the relative content of metabolites, from low (blue) to high (red). (**b**) Cluster line chart of metabolites in each cluster.

To visualize the metabolite accumulation patterns of five radial tissues, hierarchical clustering analysis (HCA) was also performed, and the relative content of differential metabolites was standardized and centralized. A clustering analysis based on the hierarchical clustering of samples (Figure 3a) indicated a clear separation of samples based on volatile metabolite differences among the sapwood, transition zone, and heartwood. It led to the creation of five clusters (Figure 3a,b). Among them, the accumulation patterns of the metabolites of clusters 1 and 2 were similar, which increased the accumulation in the SW. Additionally, the metabolites in clusters 3 and 4 also increased accumulation in the SW, while in cluster 5, the accumulation of metabolites in HW1 and HW2 was higher than that in SW1, SW2, and TZ, including *p*-ethylphenol, 2,3,4-trifluorobenzoic acid, 2,5-dichloroaniline, *p*-cymene, cadinene, and 1,3,5-triisopropylbenzene.

### 2.3. Orthogonal Partial Least Squares–Discrimination Analysis (OPLS-DA)

**Figure 4:**
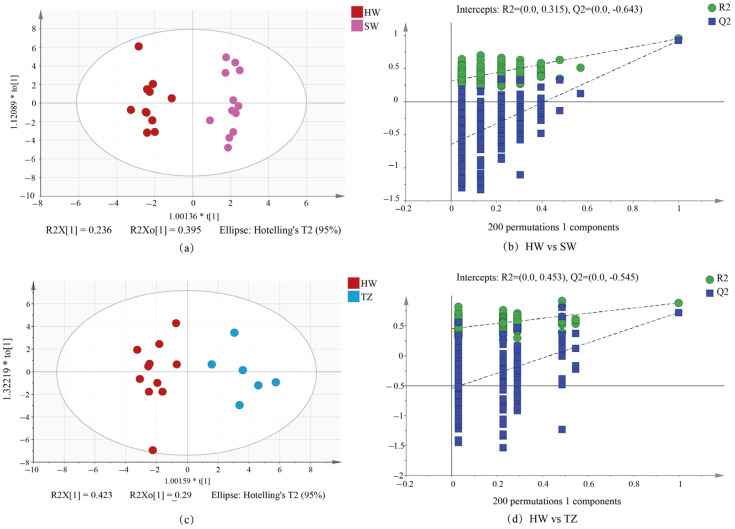
OPLS-DA of HW vs. SW and HW vs. TZ. (**a**) OPLS-DA score plot showing the discrimination of the metabolome of HW and SW. (**b**) A presentation of chance permutation at 200 times was used for the discrimination between HW and SW. (**c**) OPLS-DA score plot showing the discrimination of the metabolome of HW and TZ. (**d**) A presentation of chance permutation at 200 times was used for discrimination between HW and TZ.

In this research, the metabolic profile variations across several tissues were assessed using OPLS-DA. In particular, pairwise comparisons were specifically carried out for HW vs. SW and HW vs. TZ. The score scatter plot demonstrated that significant group distinctions were seen, indicating that there were significant differences between HW and SW and between HW and TZ (Figure 4a,c). We used 200 substitutions to verify that the OPLS-DA model was overfitted (Figure 4b,d). After inspection, Q2 < 0 and the values of R2 and Q2 (all the points on the left) were lower than the initial values of R2 and Q2 (the two rightmost points), suggesting that the model was repeatable and predictable with no noticeable overfitting.

### 2.4. Identification of Differentially Accumulated Metabolites in Pairwise Comparisons

**Figure 5:**
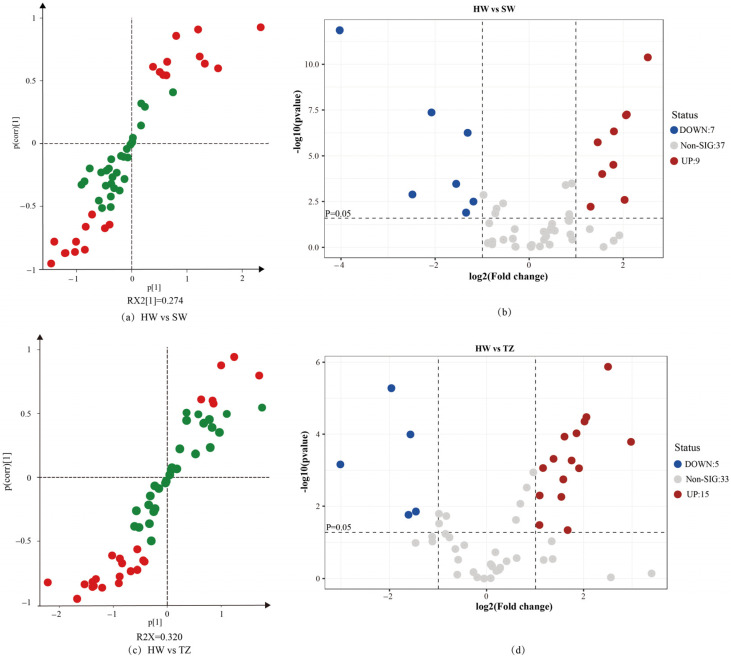
Loading S-plot diagram and volcano plot of HW vs. SW and HW vs. TZ. (**a**) The corresponding HW and SW OPLS-DA loading S-plot. The variables with VIP > 1.0 are highlighted in red, the variables with VIP < 1.0 are highlighted in blue. (**b**) A volcano map of the differential metabolites in HW and SW. Blue dots represent downregulated metabolites, red spots represent upregulated metabolites, and gray dots represent insignificant differences in metabolites. (**c**) The corresponding HW and TZ OPLS-DA loading S-plot. The variables with VIP > 1.0 are highlighted in red, the variables with VIP < 1.0 are highlighted in blue. (**d**) The volcano plot of the differential metabolites in HW and TZ. Blue dots represent downregulated metabolites, red spots represent upregulated metabolites, and gray dots represent insignificant differences in metabolites. DOWN: down-accumulated metabolite. UP: up-accumulated metabolites. Non-SIG: non-significantly different metabolites.

To determine the differential metabolites between pairwise comparisons, an OPLS-DA loading scatter plot was established. The higher values of p and p(corr) in the model were distributed in the top right and bottom left of the plot, and VIP ≥ 1 was highlighted with red dots (Figure 5a,c). Meanwhile, a volcano plot of metabolites between paired groups was also drawn to visualize the differential accumulation of secondary metabolites (Figure 5b,d). Compared with SW, 7 volatile metabolites significantly increased the accumulation in HW (FC ≥ 2, *p* < 0.05), and 15 volatile metabolites increased the accumulation in HW compared with TZ (FC ≥ 2, *p* < 0.05).

**Figure 6:**
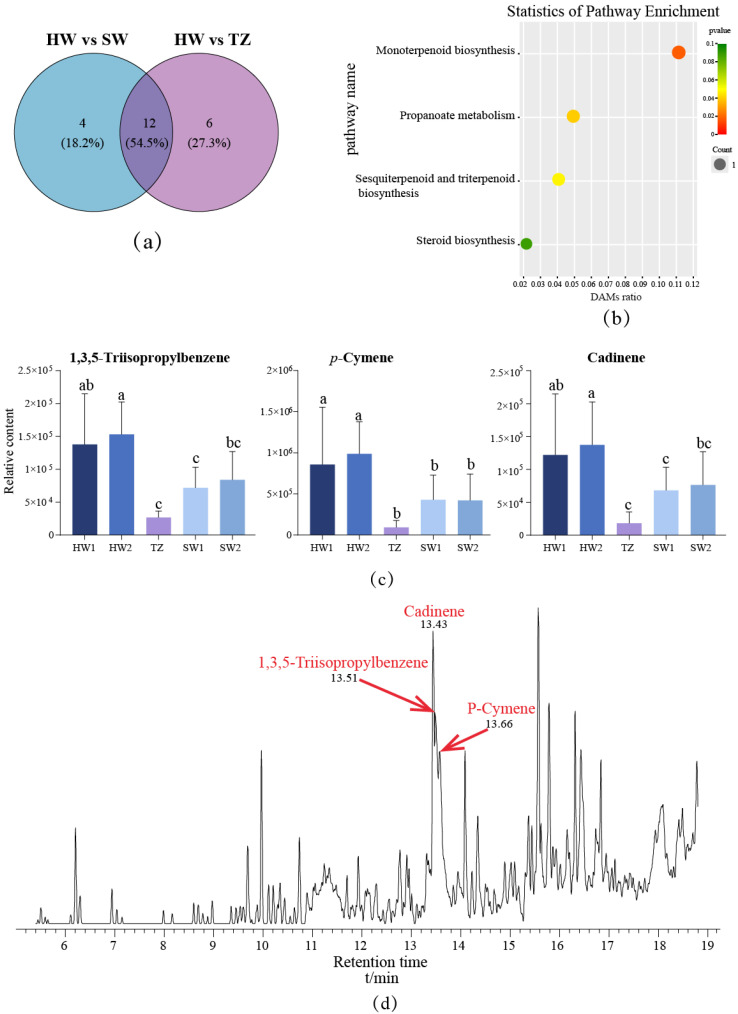
The differential accumulated metabolites related to wood fragrance. (**a**) Venn diagram of DAMs between HW and SW and between HW and TZ. (**b**) Significant accumulation of differential metabolites in HW. (**c**) The relative contents of differential accumulated metabolites in HW1, HW2, TZ, SW1, and SW2. The same letters represented difference with no significance (*p* > 0.05), different letters represented difference with significance (*p* < 0.05). (**d**) The chromatogram map displayed the three differential accumulated metabolites.

The differentially accumulated metabolites (DAMs) between pairwise comparisons were determined based on VIP ≥ 1, FC ≥ 2 or ≤0.5, and *p*-value < 0.05. Between the HW and SW group (Table S2), 16 differentially accumulated metabolites (DAMs) were identified, including 1 alkaloid, 1 aromatic hydrocarbon, 1 ester, 1 organic acid, 1 phenol, 2 organoheterocycles, 3 terpenes, and 6 other unclassified substances. In HW vs. TZ (Table S2), there were 18 DAMs, including 1 aromatic hydrocarbon, 1 phenol, 2 esters, 2 organic acids, 2 organoheterocycles, 6 terpenes, and 4 others. To screen out the unique metabolites in HW, we made Venn diagrams among different groups. Interestingly, from the Venn diagram, we found that a total of 22 DAMs were selected from the pairwise comparisons (Figure 6a), of which 12 DAMs were identified in both groups. Functional annotation analysis of 22 DAMs was carried out by using the KEGG (Kyoto Encyclopedia of Genes and Genomes) database (Figure 6b). Three metabolites were annotated in the KEGG database. Squalene in triterpenoids was annotated to sesquiterpenoid and triterpenoid biosynthesis as well as steroid biosynthesis, while monoterpenoid biosynthesis and propanoate metabolism were annotated to one significant differential metabolite, beta-myrcene and propanoic acid, respectively. In addition, combined with cluster 5 in the HCA, three metabolites with increased accumulation in HW were screened out, and a bar chart was drawn (Figure 6c). The three metabolites were cadinene, a sesquiterpenoid; *p*-cymene, a monoterpenoid; and 1,3,5-triisopropylbenzene, an aromatic hydrocarbon. The relative contents of these metabolites were significantly different from those of other tissues (*p* < 0.05). To further analyze the expression of these three specific metabolites in HW, we drew a total ion current chromatogram (Figure 6d) of HW tissue, from which we found that the relative content of these three metabolites in HW1 was significantly higher than that in other tissues (*p* < 0.05).

### 2.5. Analysis of the Key Pathway Involved in Terpenoid Biosynthesis in P. zhennan Wood

**Figure 7:**
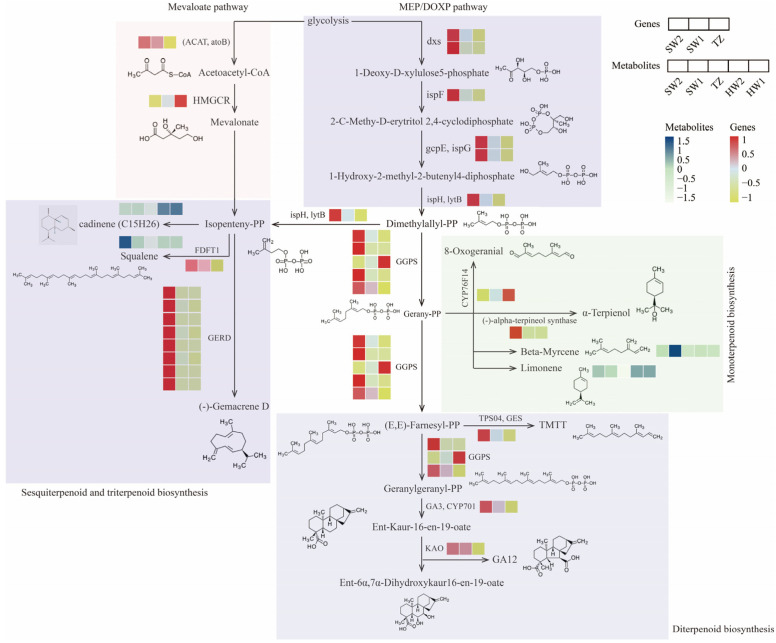
Schematic diagram of terpenoid biosynthesis pathways in *P. zhennan* wood. GERD: (-)-germacrene D synthase, TPS04, GES: geranyllinalool synthase, KAO: ent-kaurenoic acid monooxygenase, ispH, lytB: 4-hydroxy-3-methylbut-2-en-1-yl diphosphate reductase, HMGCR: hydroxymethylglutaryl-CoA reductase (NADPH), GA3, CYP701: ent-kaurene oxidase, ispF: 2-C-methyl-D-erythritol 2,4-cyclodiphosphate synthase, ACAT, atoB: acetyl-CoA C-acetyltransferase, dxs: 1-deoxy-D-xylulose-5-phosphate synthase, gcpE, ispG: (E)-4-hydroxy-3-methylbut-2-enyl-diphosphate synthase, GGPS: geranylgeranyl diphosphate synthase, type II, CYP76F14: (E)-8-carboxylinalool synthase, FDFT1: farnesyl-diphosphate farnesyltransferase. The same as below.

In total, 27 differentially expressed genes (DEGs) were screened from the RNA-seq data, which were enriched in the pathways of terpenoid backbone biosynthesis, diterpenoid biosynthesis, sesquiterpenoid and triterpenoid biosynthesis, and monoterpenoid biosynthesis (Figure 7). In terpenoid backbone biosynthesis of the MEP/DOXP pathway, two 1-deoxy-D-xylulose-5-phosphate synthase (dxs) genes (Maker00037935.gene and Phoebe_bournei_newGene_13970), one 2-C-methyl-D-erythritol 2,4-cyclodiphosphate synthase (ispF) gene (Maker00043925.gene), two (E)-4-hydroxy-3-methylbut-2-enyl-diphosphate synthases (gcpE, ispG) genes (Maker00017964.gene and Maker00051408.gene), and one 4-hydroxy-3-methylbut-2-en-1-yl diphosphate reductase (ispH, lytB) gene (Maker00015702.gene) showed significantly higher upregulation in SW2 compared with that in SW1 and TZ. In contrast, two unigenes were identified in the mevaloate pathway of terpenoid biosynthesis, of which one unigene of acetyl-CoA C-acetyltransferase (ACAT, atoB) was upregulated in sapwood more than that in TZ, and one unigene of hydroxymethylglutaryl-CoA reductase (NADPH) (HMGCR) was upregulated in TZ more than that in sapwood. A total of nine unigenes (Maker00015627.gene, Maker00056543.gene, and Maker00042781.gene, etc.) of (-)-germacrene D synthase (GERD) and farnesyl-diphosphate farnesyltransferase (FDFT1) encoding showed more than 2-fold upregulation in SW2, compared with that in SW1 and TZ. However, we detected an increased accumulation of cadiene in the HW. Upstream of monoterpenoid and diterpenoid biosynthesis, four of five unigenes encoding geranylgeranyl diphosphate synthase (GGPS) (Maker00056543.gene, Maker00018190.gene, Maker00046266.gene, and Maker00015627.gene) were upregulated in SW1; only one unigene (Maker00045458.gene) was upregulated in TZ. Further downstream, in diterpenoid biosynthesis, the unigenes encoding geranyllinalool synthase (TPS04, GES), ent-kaurene oxidase (GA3, CYP701), and ent-kaurenoic acid monooxygenase (KAO) that catalyzed the biosynthesis of TMTT, GA12, and ent-6α,7α-Dihydroxykaur16-en-19-oate were upregulated in SW more than those in TZ. In the monoterpenoid biosynthesis pathway, one unigene encoding (E)-8-carboxylinalool synthase (CYP76F14) was 2.6-fold upregulated in TZ more than that in SW2. In contrast, the unigene encoding (-)-alpha-terpineol synthase showed 1408.8 and 22.5-fold upregulation in SW2 compared to in TZ and SW1. However, we determined that the relative contents of limonene in HW were higher than in sapwood. Furthermore, the results of RT-qPCR showed that the expression patterns of the selected gene were consistent with the transcriptome data (Figure S2), which suggests that the results of the RNA-seq analysis were accurate.

### 2.6. Transcription Factors Related to Terpenoid Biosynthesis

**Figure 8:**
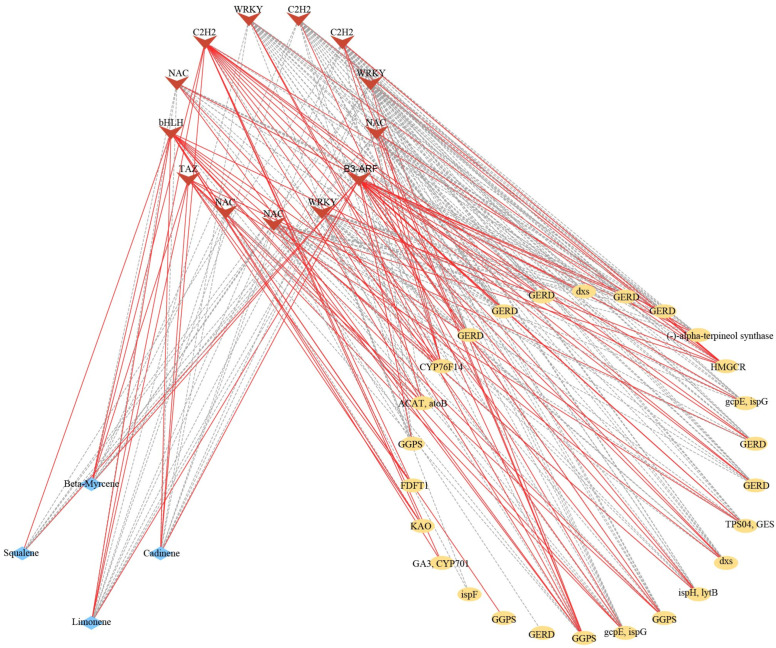
Connection network between TFs and terpenoid metabolites, pathway genes in terpenoid biosynthesis. The red v-type represents the transcription factors, the yellow diamonds represent the pathway genes in terpenoid biosynthesis, and the blue rhombus represents the terpenoid metabolites. The red solid line and grey dotted line show significant positive or negative correlations, respectively, between TFs and terpenoid, pathway genes in terpenoid biosynthesis.

There were 13 differentially expressed transcription factors (TFs) annotated to encode bHLH, C2H2, NAC, WRKY, TAZ, and B3-ARF, which were identified in the DEGs, and the expression level displayed significant correlations with the relative contents of terpenoid biosynthesis and the expression level of unigenes in terpenoid biosynthesis pathways (Figure 8). The bHLH (Maker00001762.gene) and B3-ARF (Maker00055260.gene) unigenes showed significant positive correlation with the four terpenoid metabolites. In contrast, two WRKY (Maker00014965.gene and Maker00045608.gene) and two NAC (Maker00017149.gene and Maker00017149.gene) unigenes showed significant negative correlation with the four terpenoid metabolites. Those TFs also displayed significant correlation with unigenes in the terpenoid biosynthesis pathway. For instance, the BHLH unigenes showed significant positive correlation with the expression of ent-kaurenoic acid monooxygenase (KAO) (Maker00036780.gene), ent-kaurene oxidase (GA3, CYP701) (Maker00002576.gene), and acetyl-CoA C-acetyltransferase (ACAT, atoB) (Maker00029691.gene) unigenes in the diterpenoid biosynthesis and mevaloate pathway of terpenoid backbone biosynthesis. Two WRKY unigenes (Maker00014965.gene and Maker00045608.gene) showed significant negative correlation with the almost pathway unigenes of (-)-germacrene D synthase (GERD) (Phoebe_bournei_newGene_10661, Phoebe_bournei_newGene_17052, Phoebe_bournei_newGene_3535, and Phoebe_bournei_newGene_938) in sesquiterpenoids and triterpenoid biosynthesis, (-)-alpha-terpineol synthase (Maker00000759.gene) in monoterpenoid biosynthesis, 1-deoxy-D-xylulose-5-phosphate synthase (dxs) (Phoebe_bournei_newGene_13970), (E)-4-hydroxy-3-methylbut-2-enyl-diphosphate synthase (gcpE, ispG), and 4-hydroxy-3-methylbut-2-en-1-yl diphosphate reductase (ispH, lytB) (Maker00015702.gene) in terpenoid backbone biosynthesis. In contrast, one of the two WRKY unigenes (Maker00014965.gene) displayed significant positive correlation with the unigenes of (E)-8-carboxylinalool synthase (CYP76F14) (Maker00049049.gene) and hydroxymethylglutaryl-CoA reductase (NADPH) (HMGCR) (Maker00034090.gene), and anthers WRKY unigene (Maker00045608.gene) displayed significant positive with the unigene of HMGCR.

## 3. Discussion

The characteristic odor has a great influence on the quality of wood and can improve its value. For example, *Dalbergia odorifera* heartwood releases a fragrance that has sedative and anti-inflammatory effects and is often used in traditional Chinese medicine formulations [26], and both agarwood and *Pinus cembra* are widely used in the perfumery and cosmetics industry due to their unique fragrances [27,28]. Additionally, the heartwood of *Widdringtonia cedarbergensis* has a special fragrant odor and is used to make a variety of essential oils [28]. Nanmu (*Phoebe zhennan*) is an extremely precious wood variety in China that has important application value in gardens, spices, and medicine, and the substances in its wood are worthy of further development and utilization [1]. Metabolomics analyses can allow one to analyze a sample according to the relative metabolite content and identify metabolites with special biological activities in a sample [19,20,29,30]. Fragrance related to mixtures of a variety of substances with different chemical properties can be divided into three categories according to their biosynthesis pathway: terpenoids, fatty acid derivatives, and benzenoids/phenylpropanoids [31]. In this study, comparative metabolomics using GC-MS technology was used to analyze the fragrance-related metabolites of *P. zhennan* wood and to explore the unique fragrance-related metabolites in heartwood. A total of 53 volatile metabolites belonging to 11 superclasses were detected in all tissues, and there were significant differences in the volatile metabolites among the heartwood transition zone and sapwood. Terpenoids were the most abundant substances with a special fragrance, and they are found in almost all plant flowers [32]. They can help plants resist pathogen invasion and increase wood decay resistance [33]. Among the volatile components in *P. zhennan* wood, terpenes and aromatic hydrocarbons were the main volatile components. Similar to *Phoebe hui* wood [11], among them, monoterpenes and sesquiterpenes are the representatives of terpenoids and the main sources of wood fragrance in *P. zhennan* wood.

Three volatile metabolites (cadinene, a sesquiterpenoid; p-cymene, a monoterpenoid; 1,3,5-triisopropylbenzene, an aromatic hydrocarbon) contributed heavily to the characteristic fragrance of *P. zhennan* heartwood according to the results of comparative metabolomics. The beta-myrcene was detected in monoterpenoid biosynthesis, which was an intermediate and a fragrant substance with strong anti-aging and anti-anxiety effects, and it can interact with other terpenes in plants to produce a special fragrance [34,35]. It was suggested that beta-myrcene may combine with special terpenes to endow heartwood with a unique fragrance. A study on the volatile compounds of agarwood showed that sesquiterpenes and 2-(2-phenylethyl)-4-H-chromen-4-one derivatives were the main components [36]. An analysis of the volatile compounds in cypress wood showed that the volatile oil mainly contained monoterpenes (carvacrol and *p*-cymene), sesquiterpenes (α-cedrol and cedrene), and diterpenoids (manool) [37]. In our study, eight terpenoids were identified, including three monoterpenoids (beta-myrcene, limonene, and *p*-cymene), two sesquiterpenoids (beta-gurjunene and cadinene), one triterpenoid (squalene), one 1-octadecene, and one hexadecene. Most of these substances have been identified in most essential oils of Lauraceae, giving Lauraceae plants a unique fragrance [7,38]. The terpenoids of cadinene and *p*-cymene accumulated significantly in the heartwood, while the contents of squalene, beta-myrcene, limonene, and 1-octadecene were higher than those in the sapwood. Squalene and 1-octadecene are terpenes identified in *P. zhennan* wood for the first time. Squalene is a key metabolic intermediate of sesquiterpenoid and triterpenoid biosynthesis as well as steroid biosynthesis. Studies have shown that squalene is the precursor of human synthetic vitamins, sterols, and steroids, with strong oxygen supply, anti-aging, and anti-fatigue ability, and it is widely used in the medical industry. And 1-octadecene can enhance the antibacterial and antioxidant capacity of plant extracts [39]. In this study, it was found that squalene and 1-octadecene were expressed in both HW and SW, which could be used as a source for the extraction of these two substances and provide a reference for the further development and utilization of *P. Zhennan* wood [40,41]. Additionally, it has been demonstrated that cadinene has a woody fragrance [42,43]. The pleasant fragrance of *Luculia* is due to the presence of cadinene and other volatile compounds [43]. According to Plagemann et al. [44], cadinene is one of the most prominent fragrance substances in jabuticaba fruits. *p*-cymene is also a volatile compound with a special fragrance that has been identified in many plants [45,46]. One of the main fragrance components of Nigella sativa L. seeds’ volatile essential oil is *p*-cymene [47]. Additionally, the main component in the essential oil of mature leaves of Artemisia scoparia is *p*-cymene, which has the effects of free radical scavenging and antioxidation [41]. In this study, the expression levels of cadinene and *p*-cymene in heartwood were significantly higher than those in other tissues, speculating that these two substances may be the unique fragrance components in the heartwood.

A better understanding of the regulated mechanisms by which these terpenoids are produced can help us to select and increase the production of desired compounds. Terpenoids in plants are produced by the MVA and MEP pathway using carbon sources, such as carbohydrates [21,48]. The HMGR and dxs are the key enzymes in the biosynthesis pathways of terpenoids [21]. Herein, two upregulated unigenes in dxs were observed in sapwood, and we also screened some pathway genes (e.g., ispF, gcpE, ispG, and ispH, lytB) also upregulated in sapwood in the MEP pathway. Nevertheless, the unigene encoding the key enzymes of HMGCR was upregulated in the transition zone in the MVA pathway, thereby suggesting that the MEP pathway mainly occurred in the sapwood of *P. zhennan*. GERD ((-)-germacrene D synthase) is an enzyme that catalyzes the production of (-)-gemacrene D from isopentenyl-PP in the pathway of sesquiterpenoids and triterpenoid biosynthesis [49]. The farnesyl-diphosphate farnesyltransferase (FDFT1) was the key enzyme for squalene biosynthesis, and the upregulation of FDFT1 genes may have indicated a relatively high accumulation of squalene for sesquiterpenoids in the sapwood. We also detected that almost unigenes encoding enzymes, such as GERD in sesquiterpenoids and triterpenoid biosynthesis, (-)-alpha-terpineol synthase in monoterpenoid biosynthesis, and geranylgeranyl diphosphate synthase, type II (GGPS), ent-kaurene oxidase (GA3, CYP701), and geranyllinalool synthase (TPS04, GES) in diterpenoid biosynthesis, were upregulated in the sapwood. Therefore, we boldly speculated that fragrance-related metabolites were synthesized in SW and then deposited in heartwood during sapwood’s transformation to heartwood. It is different from the wood fragrance-related metabolites in *Phoebe hui*, where terpenoid biosynthesis occurs in situ in the heartwood [11]. Numerous studies have reported transcription factors, such as AP2/ERF, WRKY, and bHLH, involved in terpenoid biosynthesis [50]. Some WRKY transcription factors transactivate the promoters of TPSs to regulate terpenoid biosynthesis [11,51,52]. *GaWRKY1* could activate the CAD1-A promoter to regulate the (+)-σ-cadinene synthase in cotton [53]. Three WRKY unigenes showed significant correlation with the expression level of pathway genes in terpenoid biosynthesis and the relative contents of terpenoids, which would positively or negatively regulate the biosynthesis of terpenoids through regulating gene expression in the terpenoid biosynthesis pathway. Chuang et al. reported *PbbHLH4* interacting with *PbbZIP4* and *PbNAC1* to regulate the biosynthesis of different monoterpenes in *Phalaenopsis orchids* [54]. The NAC transcription factor also could activate *AcTPS1* to regulate monoterpenoid biosynthesis in *Actinidia chinensis* [55]. In our study, four NAC and one bHLH transcription factors displayed significant negative correlation with the contents of beta-myrcene, squalene, limonene, and cadinene and positive or negative significant correlation with the pathway genes in terpenoid biosynthesis. C_2_H_2_ has also been reported to play an important role in enhancing terpenoid, sesquiterpenoid, and triterpenoid biosynthesis [56]. We also observed significant correlation between C_2_H_2_ and terpenoid metabolites as well as pathway genes. These results implied that those transcription factors would activate or inhibit the expression of pathway genes in terpenoid biosynthesis to regulate the synthase of fragrance-related terpenoid metabolites in *P. zhennan* wood. We will further verify these pathway genes and transcription factors and provide a more accurate theoretical basis for the breeding of *P. zhennan* and the regulation of terpenoid metabolites.

## 4. Materials and Methods

### 4.1. Plant Materials

**Figure 9:**
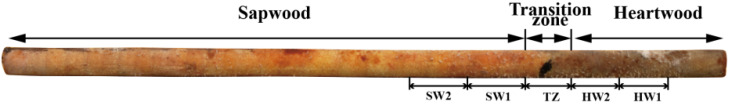
Schematic diagram of sampling and division of nanmu wood core. Transition zone (TZ), outer heartwood (HW1), inner heartwood (HW2), outer sapwood (SW1), and inner sapwood (SW2).

The samples were collected from ~80-year-old *P. zhennan* in Du Fu Thatched Cottage Museum in Chengdu, Sichuan Province, China (N 30°39′37.00″, E 104°1′41.69″). We selected the increment cores of six trees for sampling (six biological repeats). We used a growth cone (150 mm) to drill the increment core at breast height (1.3 m) per tree. Then, the increment core was divided into five tissues along the radical section according to color: the transition zone (TZ) at the junction of the dark and light colors; the heartwood part (HW2) located near the TZ; the heartwood part far away from the TZ (HW1); the outer edge of the sapwood part near the TZ (SW2); and the inner sapwood (SW1) of the sapwood section far away from the TZ (Figure 9). Further, 5–7 growth rings were included in SW1, SW2, HW1, and HW2, respectively, and 2–3 growth rings were included in TZ [2]. All samples were rapidly frozen in liquid nitrogen and stored at −80 °C for further use.

### 4.2. Sample Preparation for Metabolite Profiling

Wood samples weighing 2.0 g were weighed accurately and ground into a fine powder (MB-96, Zhejiang Meibi Experiment Equipment Co., Ltd., Zhejiang, China) in liquid nitrogen. The wood powder and 600 µL of ethyl acetate solution were put into a 2 mL centrifuge tube and swirled for 1 min (BE-96, Haimen Kylin-Bell Lab Instruments Co., Ltd., Haimen, China) and then vibrated in an ultrasonic oscillator for 10 min (KW-100TDV, Kunshan Shumei Experiment Equipment Co., Ltd., Kunshan, China). The metabolites were extracted using the Soxhlet extraction method at room temperature in the dark for 12 h. After centrifugation at 21,000× *g* and 4 °C for 10 min (H1850-R, Hunan Xiang Yi Laboratory Instrument Development Co., Ltd., Xiangtan, China), the supernatant was poured through a microporous membrane filter (0.22 µm pore size; Tianjin Jinteng Experiment Equipment Co., Ltd., Tianjin, China) and placed into an injection bottle for GC-MS analysis. Meanwhile, an equal proportion mixture of all the chemical compounds in the extraction was prepared as a quality control (QC) sample, which was injected alongside every sample in order to monitor the stability of the analysis.

### 4.3. GC-MS Analysis Conditions

All sample extracts were analyzed using an Agilent 8890A gas chromatography column equipped with a 5977B mass spectrometer (Agilent, Shanghai, China). The gas chromatographic column was an Agilent 19091S-433UI column (30 m × 0.25 mm i.d., 0.25 μm film thickness). The injection volume was 1.0 µL. The settings used for the temperature program were as follows: a starting temperature of 60 °C, increase to 160 °C at 8 °C·min^−1^, and then increase to 220 °C at 10 °C·min^−1^. The total GC runtime was 18.5 min. The carrier gas was nitrogen, which flowed at a rate of 1.0 mL·min^−1^. The quadrupole was at 150 °C, and the ion source was at 200 °C. The solvent delay time was 5.0 min. The electron impact ion energy was 70 eV. The mass scan range was *m*/*z* 50–500.

### 4.4. Metabolite Identification

We used the data preprocessing and statistical analysis software ProteoWizard version 3.0 to convert the original data file into mzXML file format. The mzXML files were processed and analyzed in MS-DIAL 4.9.221218, including peak detection and peak matching. Then, the data matrix composed of sample information, retention time (RT), the mass-to-charge ratio (*m*/*z*), and peak area was transformed by log10 and imported into the Majorbio Cloud platform (https://cloud.majorbio.com/page/tools/) (accessed on 16 March 2022) for follow-up analysis. All ions with missing values for less than 80% all samples were screened out from the dataset, and the remaining missing values were replaced with minimum values. Then, the sum method was used to normalize the data matrix to eliminate the ions with relative standard deviations (RSDs) > 30% in the quality control samples. Based on database of Majorbio Clond platform, these identified metabolites were annotated via tandem mass spectrometers, carried out based on matching the characteristics, including RT and *m*/*z* values with the Human Metabolome Database (HMDB) and the Kyoto Encyclopedia of Genes and Genomes (KEGG) database [57]. The n-Hexadecane (500 ng/μL) was charged as external standard to quantify the relative contents of metabolites. The corresponding relative metabolite contents were represented as chromatographic peak area integrals.

### 4.5. Statistical Analysis of Fragrance-Related Metabolites

To discriminate the metabolite variation among HW1, HW2, TZ, SW1, and SW2, principal component analysis (PCA) and orthogonal partial least-squares discrimination analysis (OPLS-DA) multivariate statistical studies were carried out using SIMCA-14.1 (Umetrics AB, Umea, Sweden). PCA analysis can preview the clustering among groups and find possible outliers. Then, we used OPLS-DA to filter out orthogonal variables independent of variables. OPLS-DA can enlarge the level of differences between groups to screen differential metabolites. The important parameters for evaluating OPLS-DA are R2X, R2Y, and Q2. Q2 > 0.5 indicates that the model is effective. A loading S-plot was then constructed that was based on the OPLS-DA results in order to show the contribution of variables to differences between groups. The initial screening criteria for the various metabolites were variable importance in the projection (VIP) ≥ 1, fold change (FC) ≥ 2 or ≤0.5, and *p*-value ˂ 0.05. VIP indicates the presence of significant metabolite differences among groups. Then, to study the changing pattern of these metabolites and find the most representative set of metabolites in the experiment, we carried out a hierarchical clustering analysis (HCA) of these metabolites. The HCA of metabolites from different samples was presented through the ‘pheatmap’ package in R, and metabolites with similar patterns of accumulation were grouped into a tendency profile (cluster). The cluster diagram was drawn according to the accumulation of metabolites in each cluster. In addition, SPSS statistics 26.0 (IBM Corporation, Armonk, NY, USA) was also used to assess volatile metabolites. Differences in metabolites in HW, SW, and TZ were assessed using ANOVA, and the differences in means were measured using the LSD test. A *p*-value < 0.05 was considered statistically significant.

### 4.6. Terpenoid Biosynthesis Pathway Analysis According to RNA-seq Data

The expression patterns of the terpenoid biosynthesis genes were investigated using our pre-published RNA-seq data obtained from wood samples of three tissues (SW1, SW2, and TZ) (the same batch of experimental materials as in this study) [2]. The RNA-seq data were deposited in the SRA database (https://www.ncbi.nlm.nih.gov/bioproject/898439) (accessed on 4 November 2022) under the identifier PRJNA898439. The differentially expressed genes (DEGs) among SW1, SW2, and TZ were screened in DESeq2 software (version 4.3) with a |log2(fold change| > 1 and FDR (false discovery rate) < 0.5 [58]. Then, the DEGs were subjected to KEGG (Kyoto Encyclopedia of Genes and Genomes) (https://www.kegg.jp/) (accessed on 4 November 2022) enrichment analyses to find the DEGs in terpenoid biosynthesis. Finally, 10 pathway genes and 5 transcription factors related to terpenoid biosynthesis were selected for RT-qPCR analysis, with the actin gene acting as the internal control for the normalization of gene expression (Table S3) [2]. The total RNA was isolated, and first-strand cDNA was synthesized using PrimeScriptTM RT reagent kit with gDNA Eraser (TaKaRa, Dalian, China). RT-qPCR was performed using TB Green Premix Ex TaqTM II (TaKaRa, Dalian, China) on a CFX96 Real-Time System (BIO-RAD, Hercules, CA, USA). Each sample was analyzed in three technical replicates.

### 4.7. Transcription Factor Related to Terpenoid Biosynthesis

The transcription factors (TFs) were subjected to an association analysis of differentially accumulated terpenoid. A correlation analysis was performed by calculating the Pearson correlation coefficient (PCC) among the terpenoid metabolite content, pathway genes, and transcription factor changes; the screening criterion was PCC ≤ −0.7 or PCC ≥ 0.7 (*p* < 0.05). Cytoscape version 3.10.0 (The Cytoscape Consortium, San Diego, CA, USA) was used to visualize the interaction networks among TFs, pathway genes, and terpenoids.

## 5. Conclusions

As for *Phoebe zhennan* wood, terpenes and aromatic hydrocarbons may be the main sources of the wood fragrance. The GC-MS-based metabolomics analysis revealed the significantly different metabolites among sapwood, the transition zone, and heartwood. The heartwood section has a special fragrance, which is attributed to strong volatile secondary metabolites accumulated during the heartwood formation process. Compared with the sapwood, the heartwood accumulated more sesquiterpenoids (cadinene), monoterpenoids (p-cymene), and aromatic hydrocarbons (1,3,5-triisopropylbenzene), which were responsible for its special fragrance. A total of 27 differentially expressed genes (DEGs) related to terpenoid biosynthesis pathways were screened from the RNA-seq data. Additionally, the expression of the DEGs in terpenoid biosynthesis pathways were especially upregulated in the SW, indicating that fragrance-related metabolites are synthesized in SW and then deposited in heartwood during sapwood’s transformation to heartwood. Several DEGs related to WRKY, C_2_H_2_, TAZ, B3-ARF, bHLH, and NAC showed significant correlation with the contents of terpenoids, which might be involved in fragrance-related metabolite biosynthesis. Overall, this study has significant implications for understanding the mechanism of accumulation in fragrance-related metabolites in *P. zhennan* wood, and it provides potential terpenoid pathway modifications to alter the wood fragrance in future breeding work on wood improvement.

## Data Availability

The data presented in this study are available in article and supplementary material.

## Supplementary Materials

The following supporting information can be downloaded at: https://www.mdpi.com/article/10.3390/molecules28207047/s1, Figure S1. Heat map of the expression of 10 classes of metabolites in HW, SW and TZ. Figure S2. The RT-qPCR analysis of the relative (left) and fragment per kilobase per million reads (FPKM) (right) of 10 enzyme genes and 5 transcription factors in SW1, SW2, and TZ. Table S1. The identification information of all metabolites. Table S2. Differential metabolites identified in HW vs. SW and HW vs. TZ. Table S3. Primers for RT-qPCR.

## References

[1] Zhu Y., An W., Peng J., Li J., Gu Y., Jiang B., Chen L., Zhu P., Yang H. (2022). Genetic diversity of Nanmu (*Phoebe zhennan* S. Lee. et F. N. Wei) breeding population and extraction of core collection using nSSR, cpSSR and phenotypic markers. Forests.

[2] Yang H., An W., Gu Y., Peng J., Jiang Y., Li J., Chen L., Zhu P., He F., Zhang F. (2022). Integrative Metabolomic and Transcriptomic Analysis Reveals the Mechanism of Specific Color Formation in *Phoebe zhennan* Heartwood. Int. J. Mol. Sci..

[3] Xiao J.-H., Ding X., Li L., Ma H., Ci X.-Q., van der Merwe M., Conran J.G., Li J. (2020). Miocene diversification of a golden-thread nanmu tree species (*Phoebe zhennan*, Lauraceae) around the Sichuan Basin shaped by the East Asian monsoon. Ecol. Evol..

[4] Tariq A., Pan K., Olatunji O.A., Graciano C., Li Z., Sun F., Sun X., Song D., Chen W., Zhang A. (2017). Phosphorous application improves drought tolerance of *Phoebe zhennan*. Front. Plant Sci..

[5] Gao J., Zhang W., Li J., Long H., He W., Li X. (2016). Amplified fragment length polymorphism analysis of the population structure and genetic diversity of *Phoebe zhennan* (Lauraceae), a native species to China. Biochem. Syst. Ecol..

[6] Jiao L., Lu Y., Zhang M., Chen Y., Wang Z., Guo Y., Xu C., Guo J., He T., Ma L. (2022). Ancient plastid genomes solve the tree species mystery of the imperial wood “Nanmu” in the Forbidden City, the largest existing wooden palace complex in the world. Plants People Planet.

[7] Xie J., Qi J., Huang X., Zhou N., Hu Y. (2015). Comparative analysis of modern and ancient buried *Phoebe zhennan* wood: Surface color, chemical components, infrared spectroscopy, and essential oil composition. J. For. Res..

[8] Shao H., Jiang Y., Pan F., Xie J., Qi J., Xiao H., Chen Y. (2020). Chemical composition, UV/vis absorptivity, and antioxidant activity of essential oils from bark and leaf of *Phoebe zhennan* SK Lee & FN Wei. Nat. Prod. Res..

[9] Wang X., Wang C., Zhang Q., Quan X. (2010). Heartwood and sapwood allometry of seven Chinese temperate tree species. Ann. For. Sci..

[10] Hoch G., Richter A., KÖRner C. (2003). Non-structural carbon compounds in temperate forest trees. Plant Cell Environ..

[11] Yang H., An W., Wang F., Gu Y., Guo H., Jiang Y., Peng J., Liu M., Chen L., Zhang F. (2022). Integrated Transcriptomic, Metabolomic, and Physiological Analyses Reveal New Insights into Fragrance Formation in the Heartwood of *Phoebe hui*. Int. J. Mol. Sci..

[12] Xie Y., Wang J., Yang F., Lei C. (2011). Comparative analysis of essential oil components of two *Cryptomeria* species from China. Ind. Crops Prod..

[13] Celedon J.M., Bohlmann J. (2018). An extended model of heartwood secondary metabolism informed by functional genomics. Tree Physiol..

[14] Ding W., Liping N., Xing H., Wei Z., Zhoua Q., Nong R., Chen J. (2020). Essential oil extracted from leaf of *Phoebe bournei* (Hemsl.) yang: Chemical constituents, antitumor, antibacterial, hypoglycemic activities. Nat. Prod. Res..

[15] Han X., Zhang J., Han S., Chong S.L., Meng G., Song M., Wang Y., Zhou S., Liu C., Lou L. (2022). The chromosome-scale genome of *Phoebe bournei* reveals contrasting fates of terpene synthase (TPS)-a and TPS-b subfamilies. Plant Commun..

[16] Qi J., Wei J., Liao D., Ding Z., Yao X., Sun P., Li X. (2022). Untargeted Metabolomics Analysis Revealed the Major Metabolites in the Seeds of four *Polygonatum* Species. Molecules.

[17] Mudiam M.K.R., Ch R., Saxena P.N. (2013). Gas Chromatography-Mass Spectrometry Based Metabolomic Approach for Optimization and Toxicity Evaluation of Earthworm Sub-Lethal Responses to Carbofuran. PLoS ONE.

[18] Castro-Alves V., Kalbina I., Nilsen A., Aronsson M., Rosenqvist E., Jansen M.A.K., Qian M., Öström Å., Hyötyläinen T., Strid Å. (2021). Integration of non-target metabolomics and sensory analysis unravels vegetable plant metabolite signatures associated with sensory quality: A case study using dill (*Anethum graveolens*). Food Chem..

[19] Peng D.-Q., Yu Z.-X., Wang C.-H., Gong B., Liu Y.-Y., Wei J.-H. (2020). Chemical Constituents and Anti-Inflammatory Effect of Incense Smoke from Agarwood Determined by GC-MS. Int. J. Anal. Chem..

[20] Liu Y., Zhu X., Qin X., Wang W., Hu Y., Yuan D. (2020). Identification and characterization of odorous volatile organic compounds emitted from wood-based panels. Environ. Monit. Assess..

[21] Huang Y., Xie F.-J., Cao X., Li M.-Y. (2021). Research progress in biosynthesis and regulation of plant terpenoids. Biotechnol. Biotechnol. Equip..

[22] Chen F., Tholl D., Bohlmann J., Pichersky E. (2011). The family of terpene synthases in plants: A mid-size family of genes for specialized metabolism that is highly diversified throughout the kingdom. Plant J..

[23] Celedon J.M., Chiang A., Yuen M.M.S., Diaz-Chavez M.L., Madilao L.L., Finnegan P.M., Barbour E.L., Bohlmann J. (2016). Heartwood-specific transcriptome and metabolite signatures of tropical sandalwood (*Santalum album*) reveal the final step of (Z)-santalol fragrance biosynthesis. Plant J..

[24] Yeh T.-F., Chu J.-H., Liu L.-Y., Chen S.-Y. (2020). Differential Gene Profiling of the Heartwood Formation Process in *Taiwania cryptomerioides* Hayata Xylem Tissues. Int. J. Mol. Sci..

[25] Dong Y., Zhang W., Ling Z., Li J., Bai H., Li H., Shi L. (2020). Advances in Transcription Factors Regulating Plant Terpenoids Biosynthesis. Chin. Bull. Bot..

[26] Choi C.W., Choi Y.H., Cha M.R., Kim Y.S., Yon G.H., Kim Y.K., Choi S.U., Kim Y.H., Ryu S.Y. (2009). Antitumor Components Isolated from the Heartwood Extract of Dalbergia odorifera. J. Korean Soc. Appl. Biol. Chem..

[27] Ghadiriasli R., Mahmoud M.A.A., Wagenstaller M., Van de Kuilen J.W., Buettner A. (2020). Molecular and sensory characterization of odorants in Cembran pine (*Pinus cembra* L.) from different geographic regions. Talanta.

[28] Kamatou G.P.P., Viljoen A.M., Özek T., Başer K.H.C. (2010). Chemical composition of the wood and leaf oils from the “Clanwilliam Cedar” (*Widdringtonia cedarbergensis* J.A. Marsh): A critically endangered species. S. Afr. J. Bot..

[29] Xing L., Sun L., Liu S., Zhang L., Sun J., Yang H. (2021). Metabolomic analysis of white, green and purple morphs of sea cucumber Apostichopus japonicus during body color pigmentation process. Comp. Biochem. Physiol. Part D Genom. Proteom..

[30] Zeng X., Li J.X., Lyu X., Chen J., Chen X.M., Guo S.X. (2022). Untargeted Metabolomics Reveals Multiple Phytometabolites in the Agricultural Waste Materials and Medicinal Materials of *Codonopsis pilosula*. Front. Plant Sci..

[31] Hanson J.R. (2003). Natural Products: The Secondary Metabolites.

[32] Cherri-Martin M., Jullien F., Heizmann P., Baudino S. (2007). Fragrance heritability in hybrid tea roses. Sci. Hortic..

[33] Pichersky E., Raguso R.A. (2018). Why do plants produce so many terpenoid compounds?. New Phytol..

[34] Surendran S., Qassadi F., Surendran G., Lilley D., Heinrich M. (2021). Myrcene-What Are the Potential Health Benefits of This Flavouring and Aroma Agent?. Front. Nutr..

[35] Sterba K., Cejka P., Culik J., Jurkova M., Krofta K., Pavlovic M., Mikyska A., Olsovska J. (2015). Determination of Linalool in Different Hop Varieties Using a New Method Based on Fluidized-Bed Extraction with Gas Chromatographic-Mass Spectrometric Detection. J. Am. Soc. Brew. Chem..

[36] Huo H.X., Zhu Z.X., Pang D.R., Li Y.T., Huang Z., Shi S.P., Zheng J., Zhang Q., Zhao Y.F., Tu P.F. (2015). Anti-neuroinflammatory sesquiterpenes from Chinese eaglewood. Fitoterapia.

[37] Balaban-Ucar M., Gonultas O. (2019). Volatile compounds of archaeological wood from the ancient harbor Thedosius in Istanbul. Eur. J. Wood Wood Prod..

[38] Joshi S.C., Padalia R.C., Bisht D.S., Mathela C.S. (2009). Terpenoid Diversity in the Leaf Essential Oils of *Himalayan lauraceae* Species. Chem. Biodivers..

[39] Tonisi S., Okaiyeto K., Hoppe H., Mabinya L.V., Nwodo U.U., Okoh A.I. (2020). Chemical constituents, antioxidant and cytotoxicity properties of *Leonotis leonurus* used in the folklore management of neurological disorders in the Eastern Cape, South Africa. 3 Biotech.

[40] Kumar L.R.G., Chatterjee N.S., Tejpal C.S., Vishnu K.V., Anas K.K., Asha K.K., Anandan R., Mathew S. (2017). Evaluation of chitosan as a wall material for microencapsulation of squalene by spray drying: Characterization and oxidative stability studies. Int. J. Biol. Macromol..

[41] Singh H.P., Kaur S., Mittal S., Batish D.R., Kohli R.K. (2010). In vitro screening of essential oil from young and mature leaves of Artemisia scoparia compared to its major constituents for free radical scavenging activity. Food Chem. Toxicol..

[42] Li Y.Y., Wan Y.M., Sun Z.H., Li T.Q., Liu X.F., Ma H., Liu X.X., He R., Ma Y., Li Z.H. (2017). Floral Scent Chemistry of *Luculia yunnanensis* (Rubiaceae), a Species Endemic to China with Sweetly Fragrant Flowers. Molecules.

[43] Miyazawa M., Nakashima Y., Nakahashi H., Hara N., Nakagawa H., Usami A., Chavasiri W. (2015). Volatile Compounds with Characteristic Odor of Essential Oil from *Magnolia obovata* Leaves by Hydrodistillation and Solvent-assisted Flavor Evaporation. J. Oleo Sci..

[44] Plagemann I., Krings U., Berger R.G., Marostica M.R. (2012). Volatile constituents of jabuticaba (*Myrciaria jaboticaba* (Vell.) O. Berg) fruits. J. Essent. Oil Res..

[45] Agulló L., Romero-Silva M.J., Domenech M., Seeger M. (2017). p-Cymene Promotes Its Catabolism through the p-Cymene and the p-Cumate Pathways, Activates a Stress Response and Reduces the Biofilm Formation in *Burkholderia xenovorans* LB400. PLoS ONE.

[46] Chung M., Cheng S., Lin C., Chang S. (2020). Profiling of aroma compounds released from cooking *Dendrocalamus latiflorus* shoots. BioResources.

[47] Kabir Y., Akasaka-Hashimoto Y., Kubota K., Komai M. (2020). Volatile compounds of black cumin (*Nigella sativa* L.) seeds cultivated in Bangladesh and India. Heliyon.

[48] Loreto F., Schnitzler J.-P. (2010). Abiotic stresses and induced BVOCs. Trends Plant Sci..

[49] Arimura G.-I., Huber D.P.W., Bohlmann J. (2004). Forest tent caterpillars (*Malacosoma disstria*) induce local and systemic diurnal emissions of terpenoid volatiles in hybrid poplar (*Populus trichocarpa* × *deltoides*): cDNA cloning, functional characterization, and patterns of gene expression of (−)-germacrene D synthase, PtdTPS1. Plant J. Cell Mol. Biol..

[50] Broun P. (2004). Transcription factors as tools for metabolic engineering in plants. Curr. Opin. Plant Biol..

[51] Ma D., Pu G., Lei C., Ma L., Wang H., Guo Y., Chen J., Du Z., Wang H., Li G. (2009). Isolation and Characterization of AaWRKY1, an Artemisia annua Transcription Factor that Regulates the Amorpha-4,11-diene Synthase Gene, a Key Gene of Artemisinin Biosynthesis. Plant Cell Physiol..

[52] He X., Wang H., Yang J., Deng K., Wang T. (2018). RNA sequencing on *Amomum villosum* Lour. induced by MeJA identifies the genes of WRKY and terpene synthases involved in terpene biosynthesis. Genome.

[53] Xu Y.-H., Wang J.-W., Wang S., Wang J.-Y., Chen X.-Y. (2004). Characterization of GaWRKY1, a cotton transcription factor that regulates the sesquiterpene synthase gene (+)-delta-cadinene synthase-A. Plant Physiol..

[54] Chuang Y.C., Hung Y.C., Tsai W.C., Chen W.H., Chen H.H. (2018). PbbHLH4 regulates floral monoterpene biosynthesis in *Phalaenopsis orchids*. J. Exp. Bot..

[55] Nieuwenhuizen N.J., Chen X., Wang M.Y., Matich A.J., Perez R.L., Allan A.C., Green S.A., Atkinson R.G. (2015). Natural Variation in Monoterpene Synthesis in Kiwifruit: Transcriptional Regulation of Terpene Synthases by NAC and Ethylene-Insensitive3-Like Transcription Factors. Plant Physiol..

[56] Sharma B., Seth R., Thakur S., Parmar R., Masand M., Devi A., Singh G., Dhyani P., Choudhary S., Sharma R.K. (2021). Genome-wide transcriptional analysis unveils the molecular basis of organ-specific expression of isosteroidal alkaloids biosynthesis in critically endangered Fritillaria roylei Hook. Phytochemistry.

[57] Ren Y., Yu G., Shi C., Liu L., Guo Q., Han C., Zhang D., Zhang L., Liu B., Gao H. (2022). Majorbio Cloud: A one-stop, comprehensive bioinformatic platform for multiomics analyses. iMeta.

[58] Varet H., Brillet-Guéguen L., Coppée J.-Y., Dillies M.-A. (2016). SARTools: A DESeq2- and EdgeR-Based R Pipeline for Comprehensive Differential Analysis of RNA-Seq Data. PLoS ONE.

