# Supplementary material for: Comparative Metabolomics and Transcriptome Analysis Reveal the Fragrance-Related Metabolite Formation in Phoebe zhennan Wood
Source: Molecules. 2023 Oct 12;28(20):7047. doi: 10.3390/molecules28207047 (PMC10608883; doi:10.3390/molecules28207047)
Supplement: Supplementary file 1 [file molecules-28-07047-s001.zip › Supplementary figures.pdf]

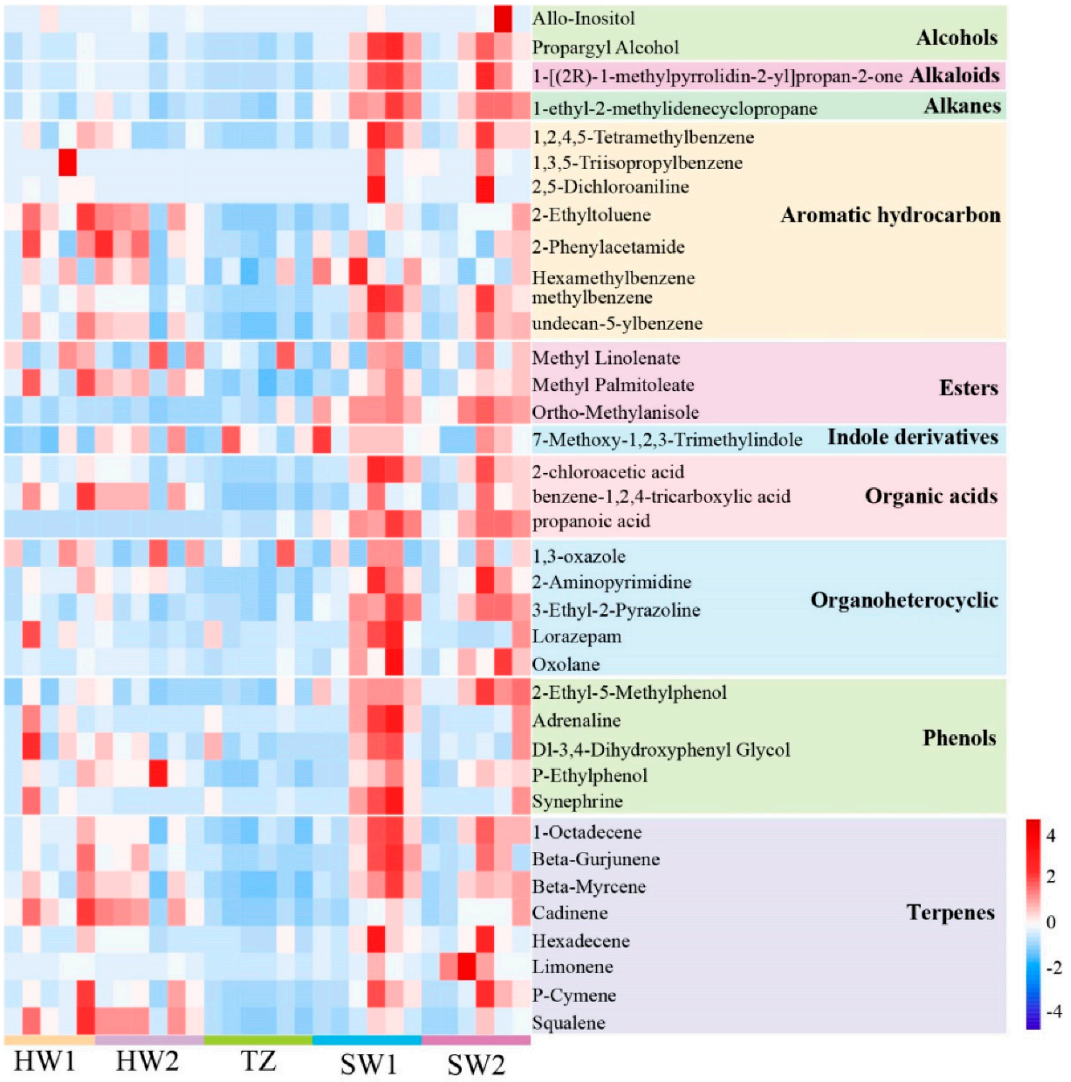

Figure S1. Heatmap of the accumulation of 10 superclasses of metabolites in HW, SW, and TZ

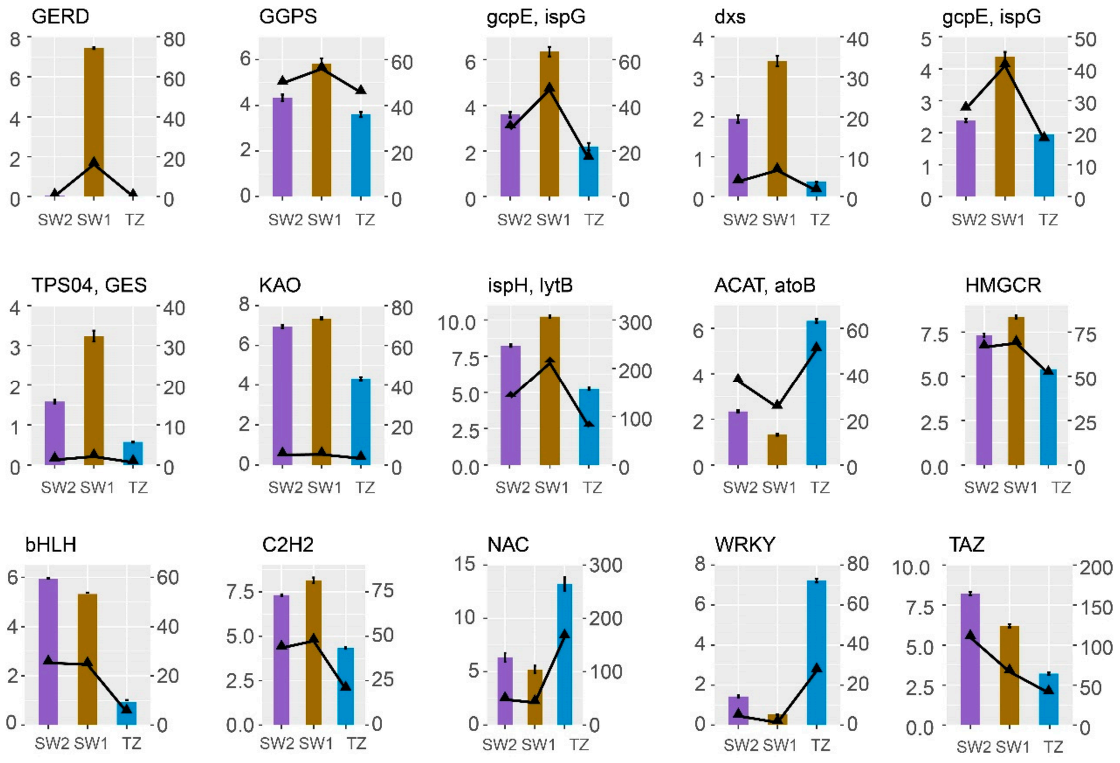

Figure S2. The RT-qPCR analysis of the relative level (left) and fragment per kilobase per million reads (FPKM) (right) of 10 enzyme genes and 5 transcription factors in SW1, SW2, and TZ.

The column showed the relative expression level, the line showed the FPKM value.
